# Predicting influenza trends in the context of post-COVID immunity gaps in Macao, China

**DOI:** 10.1093/jtm/taaf035

**Published:** 2025-04-18

**Authors:** Ying Liu, Kefeng Li, Hoiman Ng, Qianhong Ye, Henry H Y Tong

**Affiliations:** Faculty of Applied Sciences, Macao Polytechnic University, Rua de Luís Gonzaga Gomes, Macau SAR 99078, China; School of International Business, Xiamen University Tan Kah Kee College, China Merchants Zhangzhou Development Zone, Zhangzhou 363105, China; Faculty of Applied Sciences, Macao Polytechnic University, Rua de Luís Gonzaga Gomes, Macau SAR 99078, China; Clinical Laboratory, Kiang Wu Hospital, Rua de Coelho do Amaral, Santo António District, Macau SAR 99078, China; Faculty of Health Sciences and Sports, Macao Polytechnic University, Rua de Coelho do Amaral, Santo António District, Macau SAR 99078, China; Faculty of Applied Sciences, Macao Polytechnic University, Rua de Luís Gonzaga Gomes, Macau SAR 99078, China; Faculty of Health Sciences and Sports, Macao Polytechnic University, Rua de Coelho do Amaral, Santo António District, Macau SAR 99078, China

**Keywords:** Predict, influenza, Google trends, Baidu index, search term

## Abstract

We used Google and Baidu search data with machine learning to predict flu trends in Macao. ‘Cold’ was the strongest early indicator, with XGBoost showing the best performance. Findings support enhanced flu surveillance amid rising risks from post-COVID immunity debt and growing tourist inflow.

Influenza, often referred to as the flu, is a contagious respiratory infection caused by influenza viruses. The illness can manifest with a wide range of symptoms, from mild to severe, and in some instances, it can be fatal. Symptoms usually appear suddenly and may include fever, chills, cough, sore throat, nasal congestion, muscle aches, headaches, fatigue, and occasionally, diarrhoea and vomiting.^1^ According to Centers for Disease Control and Prevention (CDC) estimates for the 2023–2024 influenza season, the impact on public health was substantial. Approximately 40 million people were affected by the flu, leading to 18 million visits to healthcare providers, 470 000 hospitalizations, and 28 000 deaths attributed to influenza.^2^ These figures underscore the significant public health challenges posed by influenza during this period. As more people turn to search engines for medical information, internet search data—such as Google Trends, Baidu Index, and Twitter data—has emerged as a valuable resource for the early detection and forecasting of infectious diseases, i.e. influenza.^3–9^

Accurate forecasting of influenza is vital for public health and plays a crucial role in epidemiological research. Predicting transmission trends and peak seasons aids in preparedness, improving monitoring systems to minimize outbreak impacts. By analysing search data from Google Trends and Baidu Index, public health authorities can monitor influenza-related search trends, potentially indicating the spread of influenza.

We used search engines Google and Baidu to investigate correlations between search terms and actual influenza; forecast influenza trends using different machine learning methods and compare their prediction performance. Weekly data of positive influenza cases were obtained from Kiang Wu Hospital, Macao, China, with time period between 1 December 2019 and 5 May 2024 (see Figure S1 in Appendix). The search terms of Google Trends and Baidu Index were in Chinese and determined related to influenza. They belonged to two categories: symptoms and illness (see Figure S2 in Appendix).

Spearman correlation analysis was used to assess the association between actual influenza cases and search query data from Google Trends and Baidu Index. To evaluate the predictive power of search engine data, four modelling approaches-Extreme Gradient Boosting (XGBoost), Light Gradient Boosting Machine (LightGBM), Support Vector Machine (SVM), and Multiple Linear Regression (MLR) were implemented. Model performance was evaluated using adjusted R,^2^ Root Mean Squared Error (RMSE), Mean Absolute Error (MAE), and Mean Absolute Percentage Error (MAPE). All analyses were performed using RStudio (version 2024.09.0 + 375).

Tables S1 and S2 in Appendix presents the correlation between actual influenza and search terms from Google Trends and Baidu Index. In addition to search terms that coincided with actual flu cases, we also generated search terms that emerged 1–2 weeks prior to and 1–2 weeks after the actual flu occurrences. For Google Trends, the most correlated search terms are ‘cold’ (0.609), ‘COVID-19’ (−0.474), ‘influenza’ (0.396) and ‘cough’ (0.317). For Baidu Index, the top related terms are ‘COVID-19’ (−0.493), ‘fever’ (0.464), ‘cold’ (0.427) and ‘cough’ (0.394). Amongst these, ‘cold’ from Google Trends shows the strongest correlation, highlighting its potential as an early indicator of influenza activity. The strong association suggests that monitoring search term for ‘cold’ can provide valuable insights into disease prevalence and public concern, supporting timely public health responses and information dissemination. Identifying these most related search terms, we used them as predictors in the forecasting analysis to predict the epidemic trend of influenza.

The number of positive influenza cases was used as response variable, and those most related search terms were used as predictor variables to fit different models. Figure 1 shows evaluation metrics to evaluate model prediction performance for Google Trends respectively Baidu Index (all the values were in Tables S3 and S4 in Appendix).

**Figure 1:**
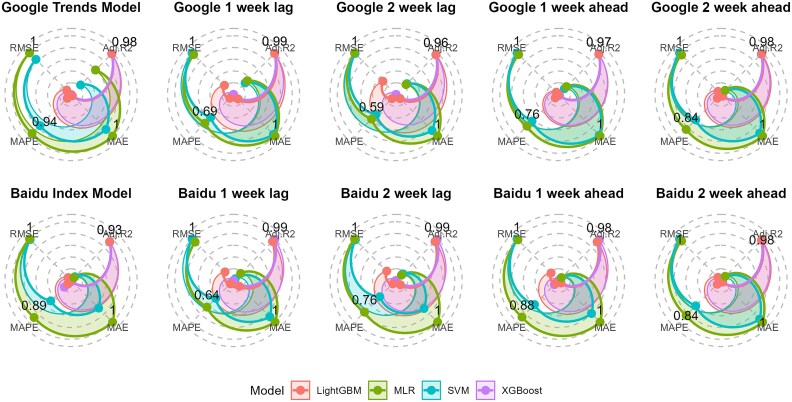
Prediction performance for models with Google Trends and Baidu Index data

Both the 1-week lag datasets from the Baidu Index and Google Trends show strong predictive capabilities for flu cases, with the XGBoost model emerging as the most effective. This integration of data enhances the accuracy and responsiveness of flu outbreak predictions, providing public health officials with a valuable tool to monitor seasonal flu trends. However, the effectiveness of predictive models may vary depending on the specific characteristics of the datasets, underscoring the importance of careful model selection in influenza prediction.

Whilst search engine data from Google and Baidu can supplement influenza surveillance, their effectiveness in Macao is constrained by behavioural, demographic, and platform-related factors. For more accurate forecasting, these tools should be used alongside traditional epidemiological data and local health monitoring systems.

Macao faces a heightened risk of influenza resurgence due to the Immunity Debt effect—reduced population immunity from limited virus circulation during COVID-19. With the easing of preventive measures and increased tourist inflow, diverse influenza strains may enter, raising transmission risks. To address this, the Macao government is boosting influenza vaccination rates through free vaccines distribution, targeted awareness campaigns, and enhanced promotion at entry points like airports, ports and ground crossings during peak flu seasons.

## Supplementary Material

Appendix_JTM_25_139_taaf035

## Data Availability

The datasets were derived from sources in the public domain: https://index.baidu.com/v2/index.html#/  https://trends.google.com/trends/explore?date=today5-y&geo=MO&q=influenza&hl=en-GB.
