# Supplementary material for: Predicting influenza trends in the context of post-COVID immunity gaps in Macao, China
Source: J Travel Med. 2025 Apr 18;32(5):taaf035. doi: 10.1093/jtm/taaf035 (PMC12210012; doi:10.1093/jtm/taaf035)
Supplement: Appendix_JTM_25_139_taaf035 [file appendix_jtm_25_139_taaf035.docx]

**Appendix**


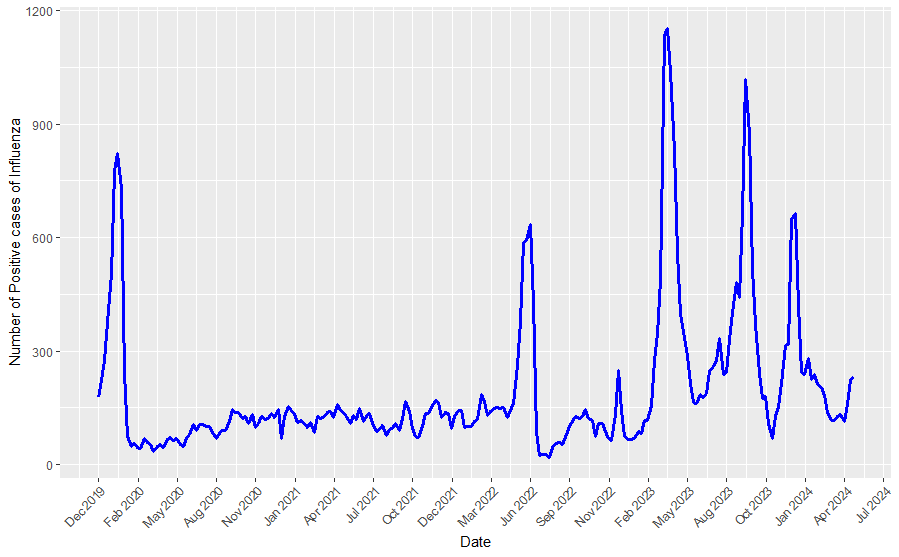


Fig S1. Weekly number of influenza positive cases in Macao, China.


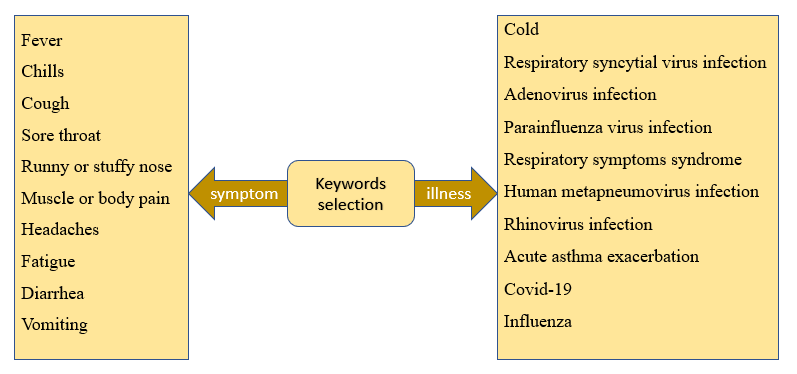


Fig S2. Google Trends and Baidu Index search terms.

Table S1. Correlation coefficients for Google Trends search term.

| search terms | -2 week | -1 week | 0 week | +1 week | +2 week |
| --- | --- | --- | --- | --- | --- |
| Fever | 0.026 | 0.091 | 0.082 | 0.044 | -0.022 |
| Cough | 0.363 | 0.348 | 0.317 | 0.243 | 0.155 |
| sore throat | -0.017 | -0.045 | -0.019 | 0.006 | 0.029 |
| stuffy nose | 0.109 | 0.173 | 0.154 | 0.106 | 0.039 |
| muscle pain | 0.055 | 0.077 | 0.050 | 0.029 | -0.004 |
| body pain | 0.017 | -0.011 | -0.002 | -0.002 | -0.005 |
| Headaches | 0.074 | 0.078 | 0.084 | 0.066 | 0.071 |
| fatigue | 0.089 | 0.086 | 0.080 | 0.060 | 0.046 |
| diarrhea | 0.040 | 0.005 | 0.072 | 0.079 | 0.061 |
| Cold | 0.584 | 0.664 | 0.609 | 0.061 | 0.078 |
| Respiratory syncytial virus infection | 0.073 | 0.025 | -0.078 | -0.091 | -0.092 |
| Parainfluenza virus infection | 0.100 | 0.101 | 0.102 | 0.093 | -0.068 |
| Respiratory symptoms syndrome | -0.005 | 0.017 | 0.021 | -0.011 | 0.043 |
| Human metapneumovirus infection | -0.087 | -0.065 | -0.079 | -0.022 | 0.009 |
| Acute asthma exacerbation | -0.085 | -0.001 | 0.038 | 0.060 | 0.081 |
| COVID-19 | -0.427 | -0.468 | -0.474 | -0.496 | -0.493 |
| Influenza | 0.408 | 0.429 | 0.396 | 0.345 | 0.245 |

Note: Search term “chills”, “Adenovirus infection” and “Rhinovirus infection” were not found in Google Trends. “vomiting” does not have enough valid data to calculate the correlation coefficient.

Table S2. Correlation coefficients for Baidu Index search term.

| search terms | -2 week | -1 week | 0 week | +1 week | +2 week |
| --- | --- | --- | --- | --- | --- |
| Fever | 0.521 | 0.519 | 0.464 | 0.315 | 0.260 |
| Cough | 0.463 | 0.452 | 0.394 | 0.295 | 0.217 |
| sore throat | 0.114 | 0.047 | 0.017 | -0.035 | -0.051 |
| stuffy nose | 0.086 | 0.099 | 0.071 | 0.051 | 0.017 |
| muscle pain | -0.010 | -0.078 | -0.067 | -0.058 | -0.067 |
| Headaches | -0.195 | -0.218 | -0.238 | -0.249 | -0.250 |
| fatigue | 0.027 | 0.002 | -0.011 | -0.038 | -0.069 |
| diarrhea | 0.116 | 0.146 | 0.096 | 0.074 | 0.020 |
| vomiting | 0.119 | 0.151 | 0.161 | 0.130 | 0.112 |
| Cold | 0.429 | 0.449 | 0.427 | 0.351 | 0.226 |
| Adenovirus infection | 0.128 | 0.108 | 0.142 | 0.142 | 0.140 |
| COVID-19 | -0.479 | -0.490 | -0.493 | -0.458 | -0.425 |
| Influenza | 0.259 | 0.282 | 0.222 | 0.115 | 0.022 |

Note: Search term “chills”, “body pain”, “Respiratory syncytial virus infection”, “Parainfluenza virus infection”, “Respiratory symptoms syndrome”, “Human metapneumovirus infection”, “Rhinovirus infection” and “Acute asthma exacerbation” were not found in Baidu Index.

Table S3. Prediction performance for Google Trends Models.

| **Model** | **Adjusted R²** | **MAE** | **MAPE** | **RMSE** |
| --- | --- | --- | --- | --- |
| **Baidu Index Data** |  |  |  |  |
| SVM | 0.23 | 109.54 | 74.93% | 178.11 |
| XGBoost | 0.98 | 14.64 | 8.87% | 30.11 |
| LightGBM | 0.98 | 18.14 | 9.15% | 48.54 |
| MLR | 0.59 | 127.47 | 94.02% | 205.20 |
| **Baidu Lagged 1 Week** |  |  |  |  |
| SVM | 0.28 | 103.12 | 55.41% | 181.03 |
| XGBoost | 0.99 | 9.59 | 4.81% | 19.61 |
| LightGBM | 0.99 | 20.08 | 8.83% | 55.19 |
| MLR | 0.33 | 107 | 69.15% | 174.83 |
| **Baidu Lagged 2 Weeks** |  |  |  |  |
| SVM | 0.26 | 75.62 | 47.72% | 135.81 |
| XGBoost | 0.96 | 17.54 | 10.38% | 31.51 |
| LightGBM | 0.96 | 24.09 | 10.35% | 65.13 |
| MLR | 0.24 | 84.40 | 58.85% | 137.18 |
| **Baidu Ahead 1 Week** |  |  |  |  |
| SVM | 0.14 | 137.18 | 64.2% | 226.44 |
| XGBoost | 0.97 | 21.15 | 8.28% | 40.01 |
| LightGBM | 0.97 | 18.52 | 7.86% | 50.23 |
| MLR | 0.19 | 139.77 | 76.13 | 219.39 |
| **Baidu Ahead 2 Weeks** |  |  |  |  |
| SVM | 0.02 | 117.8 | 60.69% | 222.21 |
| XGBoost | 0.97 | 15.16 | 7.95% | 35.82 |
| LightGBM | 0.98 | 19.48 | 8.66% | 49.81 |
| MLR | 0.1 | 125.46 | 83.87% | 213.19 |

*The MAE and RMSE values ​​in Figure 1 are normalized. The original values ​​are shown here.

Table S4. Prediction performance for Baidu Index Models.

| **Model** | **Adjusted R²** | **MAE** | **MAPE** | **RMSE** |
| --- | --- | --- | --- | --- |
| **Baidu Index Data** |  |  |  |  |
| SVM | 0.09 | 76.28 | 49.34% | 153.95 |
| XGBoost | 0.93 | 20.80 | 15.82% | 41.84 |
| LightGBM | 0.93 | 18.93 | 8.04% | 49.75 |
| MLR | 0.07 | 105.23 | 89.19 | 155.64 |
| **Baidu Lagged 1 Week** |  |  |  |  |
| SVM | 0.04 | 84.57 | 42.64% | 174.11 |
| XGBoost | 0.994 | 7.17 | 4.11% | 14.31 |
| LightGBM | 0.94 | 19.40 | 8.37% | 47.17 |
| MLR | 0.17 | 95.73 | 63.50 | 162.25 |
| **Baidu Lagged 2 Weeks** |  |  |  |  |
| SVM | 0.16 | 70.04 | 39.29% | 152.79 |
| XGBoost | 0.99 | 8.97 | 6.14% | 16.68 |
| LightGBM | 0.939 | 18.29 | 7.51% | 47.99 |
| MLR | 0.13 | 99.61 | 75.55 | 155.70 |
| **Baidu Ahead 1 Week** |  |  |  |  |
| SVM | 0.04 | 85.91 | 58.26% | 172.21 |
| XGBoost | 0.98 | 13.08 | 10.08% | 23.27 |
| LightGBM | 0.93 | 18.68 | 7.02% | 50.11 |
| MLR | 0.07 | 104.81 | 88.46 | 169.23 |
| **Baidu Ahead 2 Weeks** |  |  |  |  |
| SVM | 0.02 | 87.10 | 56.52% | 149.31 |
| XGBoost | 0.97 | 16.42 | 13.09% | 27.15 |
| LightGBM | 0.93 | 16.96 | 6.26% | 49.62 |
| MLR | 0.07 | 104.99 | 93.43 | 145.51 |

*The MAE and RMSE values ​​in Figure 1 are normalized. The original values ​​are shown here.
